# Morphological and functional assessment of the left atrial appendage in daily practice: a comprehensive approach using basic and advanced echocardiography with practical tips

**DOI:** 10.1186/s44348-024-00017-2

**Published:** 2024-07-29

**Authors:** Ashraf M. Anwar

**Affiliations:** 1 Department of Cardiology, King Fahad Armed Forces Hospital, Jeddah, Saudi Arabia; 2 Department of Cardiology, Faculty of Medicine, Al-Azhar University, Cairo, Egypt

**Keywords:** Left atrial appendage, Functional assessment, Daily practice, Echocardiographic modalities

## Abstract

Cardioembolic stroke is the most serious and life-threatening complication of atrial fibrillation (AF), with an associated mortality up to 30% at 12 months. Approximately 47% of thrombi in valvular AF and 91% of thrombi in nonvalvular AF are localized in the left atrial appendage (LAA). Therefore, identification or exclusion of LAA thrombi is critical in many clinical situations. It is essential to assess LAA morphology and function using imaging modalities (particularly echocardiography) before, during, and after interventional procedures such as AF ablation and LAA occlusion. This review article describes the anatomical, physiological, and pathological background of the LAA, followed by an assessment of different echocardiographic modalities. Many practical points are included to improve the diagnostic accuracy and to minimize errors during image acquisition and interpretation. In each clinical scenario where LAA is the crucial target, specific and essential information and parameters are collected.

## Anatomical and histological background

The left atrial appendage (LAA) is a remnant of the original embryonic left atrial (LA) that develops during the third week of gestation. The LAA is a long, tubular, hooked structure that lies within the fixed, relatively immobile confines of the pericardium. It is usually crenellated and has a narrow junction with the venous component of the LA. There are considerable variations in its volume (0.7–19.2 mL), shape, number of lobes, and relationships with adjacent cardiac structures. The LAA is trabeculated, with pectinate muscle bars largely running parallel to each other. Its orifice has few cardiomyocytes, while the body of the LAA is rich in cardiomyocytes [1, 2].

## Echocardiographic assessment of LAA morphology

Using echocardiography, it is important to identify the complex LAA anatomic features and structure, which include the following.

### LAA pectinate muscles

**Fig. 1:**
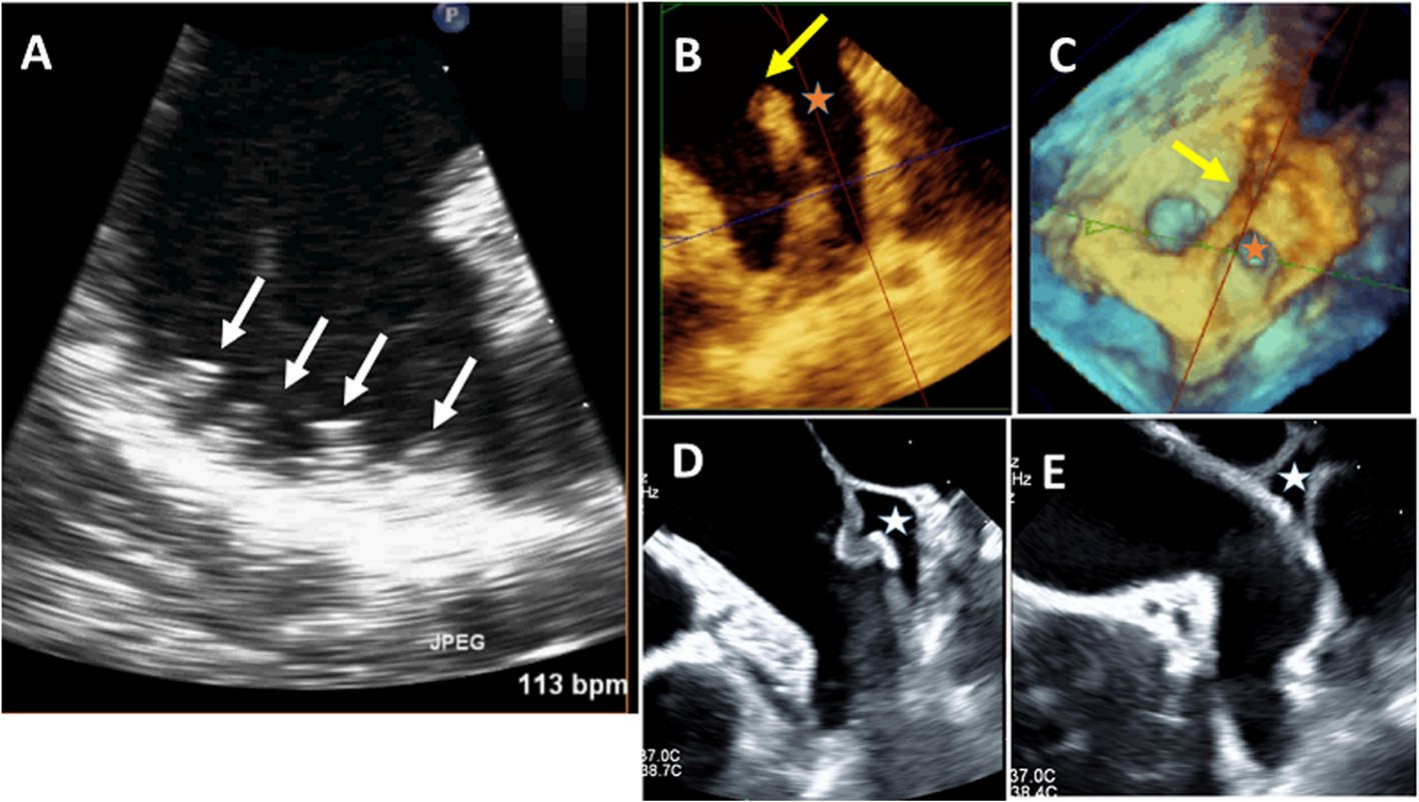
Normal left atrial appendage (LAA) anatomical structures as visualized by two dimensional (2D) transesophageal echocardiography (TEE). **A**Thick pectinate muscle at the apex of the LAA (white arrows), prominent coumadin ridge (yellow arrow) between the LAA and left upper pulmonary vein (red star) as seen by (**B**) 2D-TEE and (**C**) 3D-TEE *en face* view. **D**, **E** Transverse pericardial sinus (white star) as visualized by 2D-TEE

Rigid muscle bundles that line the LAA cavity and are arranged in a feather-type-palm-leaf pattern, especially at the borders between its superior and inferior surfaces. The thicker pectinate muscles may be mistaken for thrombi or intra-atrial masses. In atrial fibrillation (AF), structural remodeling of the LAA occurs involving dilation and reduction in the number of pectinate muscles (Fig. 1A) [3].

### Coumadin ridge

The coumadin ridge is a normal variant of the LA that encompasses the ligament of Marshall. It represents the confluence of the long axis of the left upper pulmonary vein with the roof of the LAA. Using both transthoracic echocardiography (TTE) and transesophageal echocardiography (TEE), it appears as a nodular, pedunculated, or linear structure in the wall of the LA between the left upper pulmonary vein and the LAA. Usually, it is attached to the roof of the LAA with a rounded end (75%) that extends into the LA. While the coumadin ridge is often called the “Q tip” sign, it can also be flat (15%) or pointed (10%). Its significance originated in the early days of TEE when it was mistaken for a thrombus or tumor. However, it has a different echogenicity from that of a tumor and moves with the underlying tissue (Fig. 1B, C) [4, 5].

### Transverse pericardial sinus

The transverse sinus is the pericardial reflection superior to the pulmonary veins and posterior to the arterial trunk. In a small proportion of individuals, the tip of the LAA lies within the transverse sinus. This sinus may contain fluid with or without echo-dense fibrinous material and can be mis-interpreted as LAA thrombi (Fig. 1D, E) [6].

### LAA lobes

**Fig. 2:**
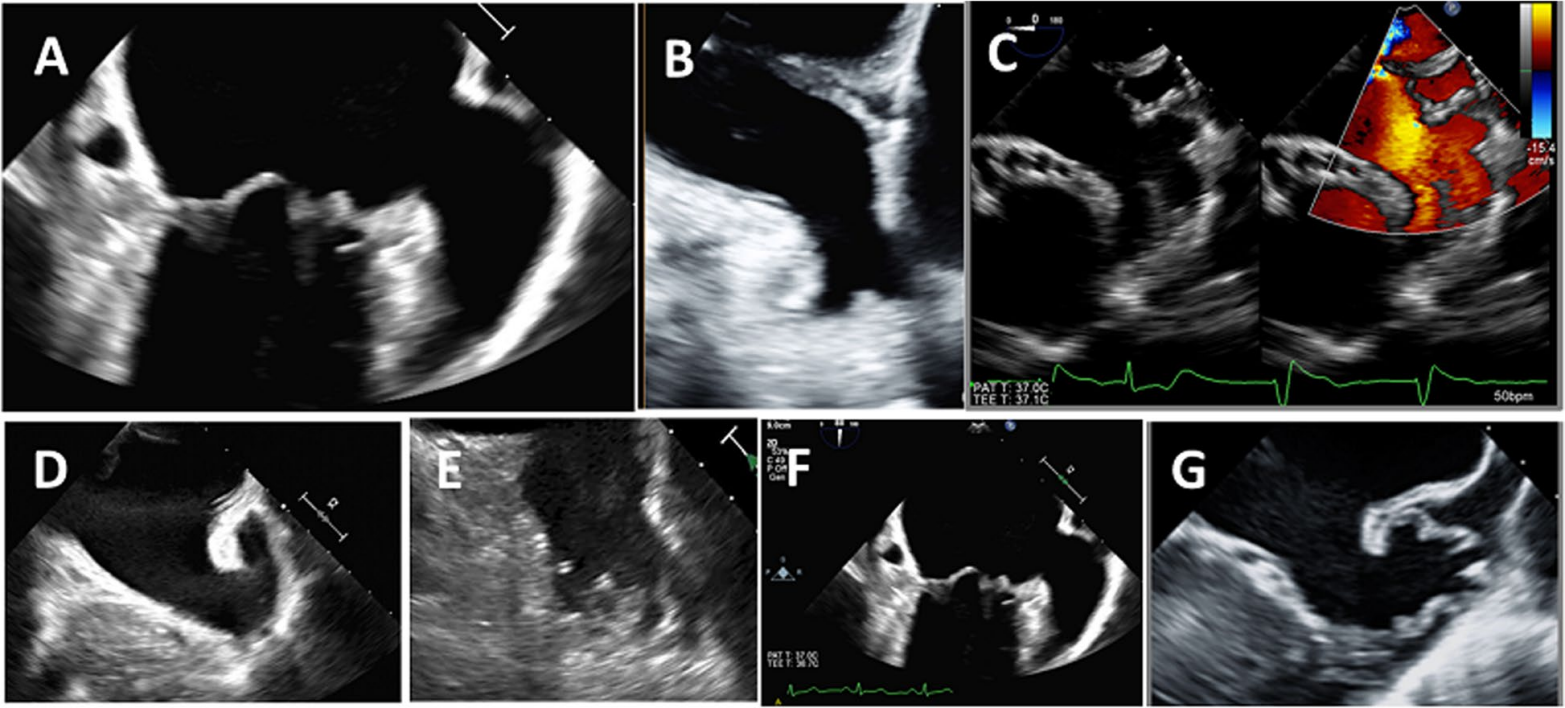
Upper panel shows two-dimensional transesophageal echocardiography examples of the left atrial appendage (LAA) lobe. **A** Single-lobed, (**B**) bi-lobed, an (**C**) tri-lobed LAA with color Doppler flow inside. Lower panel shows different LAA morphology of (**D**) chicken wing, (**E**) cactus, (**F**) windsock, and (**G**) cauliflower

Among 500 human anatomic specimens, two lobes were most common (54%) (Fig. 2B), followed by three lobes (23%) (Fig. 2C), one lobe (20%) (Fig. 2A), and four lobes (3%) [7]. The number of LAA lobes is an independent risk factor of thromboembolic events as evidenced by many studies. The anatomical definition of an LAA lobe comprise the following criteria [7, 8]:

- At least one lobe visualized as an outpouching from the body of the LAA with a minimum inner diameter of 2 mm and that was demarcated partially by an external crease.
- The anatomic plane of the lobe occasionally lies in the same anatomic plane as the main tubular body of the LAA or can be in the plane opposite that of the main tubular body.

### LAA morphology

Similar to computed tomography and magnetic resonance descriptions of LAA morphology, it was identified on TEE as follows [9]:

- A chicken wing pattern with a dominant lobe showing an obvious bend in its proximal or middle part (Fig. 2D).
- The cactus LAA pattern has a dominant central lobe and secondary lobes that arise from it superiorly and inferiorly (Fig. 2E).
- The windsock pattern has a dominant lobe with variations in the location and number of lobes (Fig. 2F).
- The cauliflower pattern has a short overall length, a variable number of lobes with lack of a dominant lobe, and an irregular orifice shape (Fig. 2G).

### LAA depth

The LAA depth is defined as the longest distance measured from the orifice at the circumflex artery to lobe tip at left ventricular (LV) end-systole. The loading condition and LA pressure may alter the LAA depth and orifice diameter, which is relevant clinically when considering the appropriate device size for LAA closure [10].

### LAA orifice

The site of reflection of the LAA with the surrounding LA wall. Most LAAs have a well-defined orifice that leads to a neck region that opens to the LAA body. The orifice shape is usually oval (81.5%), although triangular (7.3%), semicircular (4.0%), water drop-like (3.2%), round (2.4%), and foot-like (1.6%) shapes are also observed [11]. Systolic measurements tend to be 15% to 20% higher than diastolic ones. Using two dimensional (2D)-TEE, the measurement must be performed from the top of the limbus to the muscular band at the mitral valve.

### LAA neck

The neck is the narrowest part of the LAA and typically is located just distal to the ostium. The neck usually overlies the circumflex coronary artery and has a highly variable width. Neck dilation has been associated with an increased risk for stroke [12]. It is crucial to measure accurately the LAA neck because it is the landing zone for closure devices. There is wide anatomic variation in the distance between the ostium and the neck of the LAA. A considerable neck length is favorable for endovascular closure, while a short neck (e.g., chicken-wing LAA) can prevent the use of longer devices, such as the Watchman (Boston Scientific) or the Amplatzer Cardiac Plug (Abbott).

## Physiological background

Normally, the LAA makes up approximately 10% of the entire LA volume. The LAA has greater shortening and stronger contraction than does the rest of the LA. The pattern of contraction is distinct and contains four phases with constant sequences:

1. Early LAA emptying phase: forward flow occurring during the early part of ventricular diastole.
2. Short phase of backward flow into the LAA.
3. Late LAA emptying: second phase of forward flow due to active LAA contraction coincident with atrial systole.
4. Another phase of backward flow possibly caused by the combined effects of LAA relaxation and elasticity.

The magnitude of LAA emptying and filling may be related inversely to the ventricular rate and is influenced more by changes in the LV than by LAA function [13].

### Echocardiographic assessment of LAA function

Function must be evaluated as part of the standard echocardiographic examination of the LAA because it provides incremental information about the risk of clot formation, embolic events, success of cardioversion, etc. The following echocardiographic parameters can be used as indicators of LAA contractile function.

#### LAA flow velocity

**Fig. 3:**
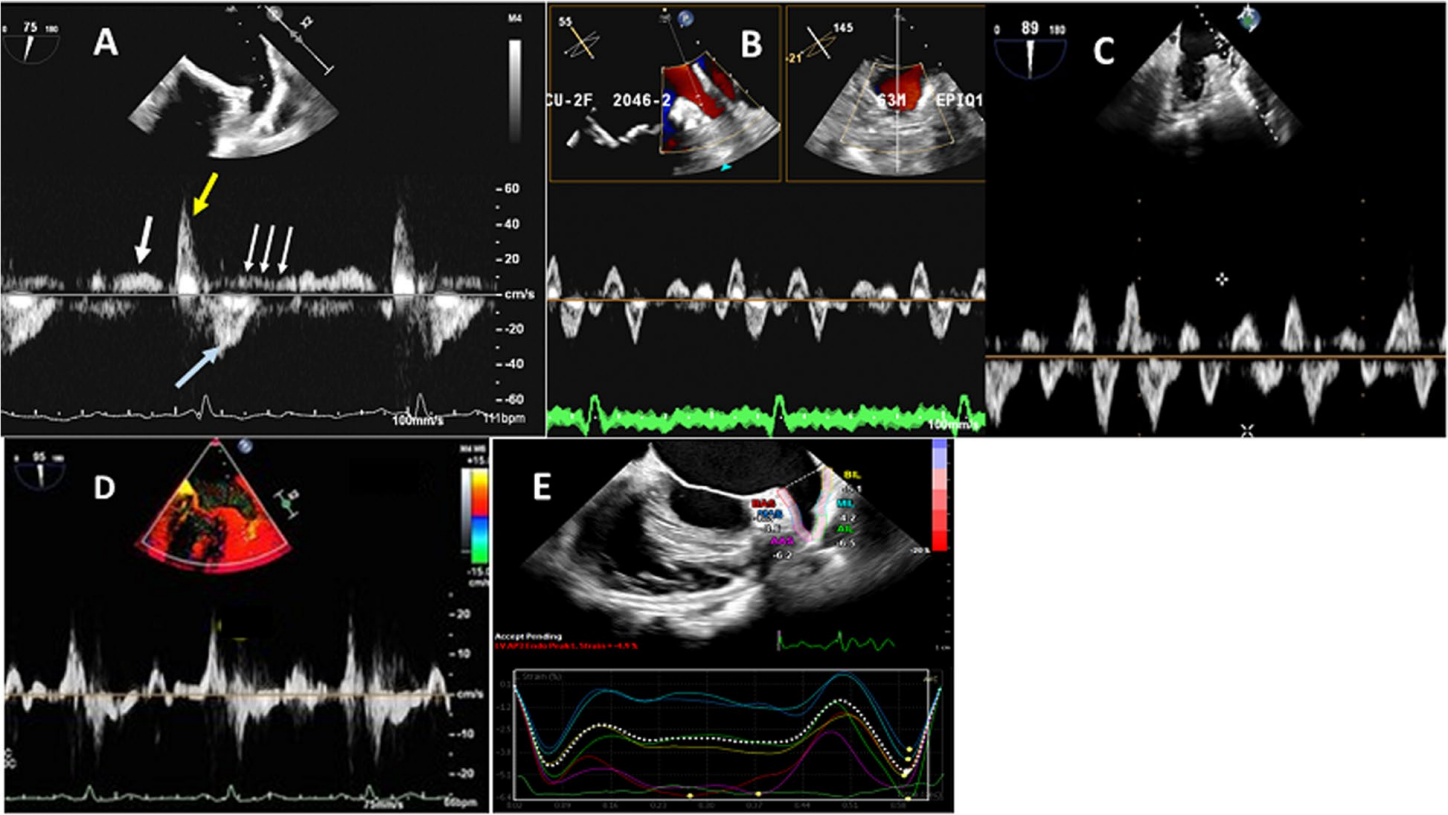
Pulsed wave Doppler of the left atrial appendage (LAA) flow velocity pattern. **A** In normal sinus rhythm (white thick arrow points toward the early diastolic emptying velocity, yellow thick arrow points toward the late diastolic emptying velocity, blue thick arrow points toward the LAA filling velocity, the three small arrows point toward the systolic reflection waves. **B** In patients with Atrial fibrillation. **C** In patients with atrial flutter, the lower panel shows (**D**) the tissue velocity pattern of the LAA as obtained by tissue-Doppler imaging and (**E**) longitudinal strain values and curves of the LAA measured by speckle tracking echocardiography

Using pulsed wave Doppler, the quadriphasic flow pattern is detected easily in patients with sinus rhythm and a slow heart rate (Fig. 3A) [14, 15].

1. Early diastolic emptying velocity: Low-velocity outflow signal is seen immediately after the mitral inflow E wave and correlates with the mitral E and pulmonary vein diastolic velocities (Fig. 3A, white arrow). The average velocity range is 20 to 40 cm/sec.
2. Late diastolic emptying velocity (LAA contraction flow): The LAA contraction flow correlates with the LAA ejection fraction and LA size and pressure and is a significant predictor of thromboembolic risk (Fig. 3A, yellow arrow). The average velocity is 50 to 60 cm/sec.
3. LAA filling velocity: A negative wave occurs immediately following LAA contraction and correlates well with the LAA contraction velocity (Fig. 3A, blue arrow). The average LAA filling velocity is 40 to 50 cm/sec.
4. Systolic reflection waves: Low velocity, multiple, alternate inflow-outflow waves follow the more prominent filling wave described above and usually are seen in patients with a slow heart rate (Fig. 3A, small white arrows). Their functional significance is not clear.

In patients with AF, saw-tooth waves of varying regularity or markedly reduced or nearly absent waves can be seen (Fig. 3B). In atrial flutter, the saw-tooth waves are more regular and have a greater amplitude than those in AF but lower amplitude than that in sinus rhythm (Fig. 3C).

#### LAA tissue velocity

Using color-coded tissue-Doppler imaging, the LAA tissue Doppler velocities can be recorded from the lateral and/or medial walls of the LAA. The profile is triphasic: S and E occur during LV contraction and relaxation, respectively, and A occurs after LA contraction (Fig. 3D) [16].

#### LAA volume and emptying fraction

Using 2D-TEE, the LAA endocardium is traced at some angles of probe rotation (0°, 45°, 90°, 135°) to obtain the average LAA volumes at end-diastole (LAA vol_max_) and end-systole (LAA vol_min_). The LAA volume and emptying fraction (LAA-EF) is calculated as follows: “(average LAA vol_max_ – average LAA vol_min_) / average LAA vol_max_.” The LAA-EF also can obtained by speckle-tracking echocardiography (STE) and by 3D-TEE. An impaired LAA-EF (< 40%) predicts paroxysmal AF after cryptogenic stroke [17] and also short- and long-term outcomes post stroke [18]. Increased LAA volume and LAA-EF < 44% have the highest value for predicting AF recurrence following radiofrequency ablation [19].

#### LAA area change

Using the previous tracing, the LAA area changes are calculated using the formula “(LAA area_max_ – LAA area_min_) / LAA area_max_ × 100.”

## Pathological background

The anatomic structure of the LAA and dilatation of the LA or LAA in valvular and nonvalvular heart disease allow erythrocyte Rouleau formation due to blood stasis. This disease starts with spontaneous echo contrast (SEC), progresses to sludge formation, and results in thrombus formation. These stages of blood stasis can be identified using TEE, as follows.

### Spontaneous echo contrast

**Fig. 4:**
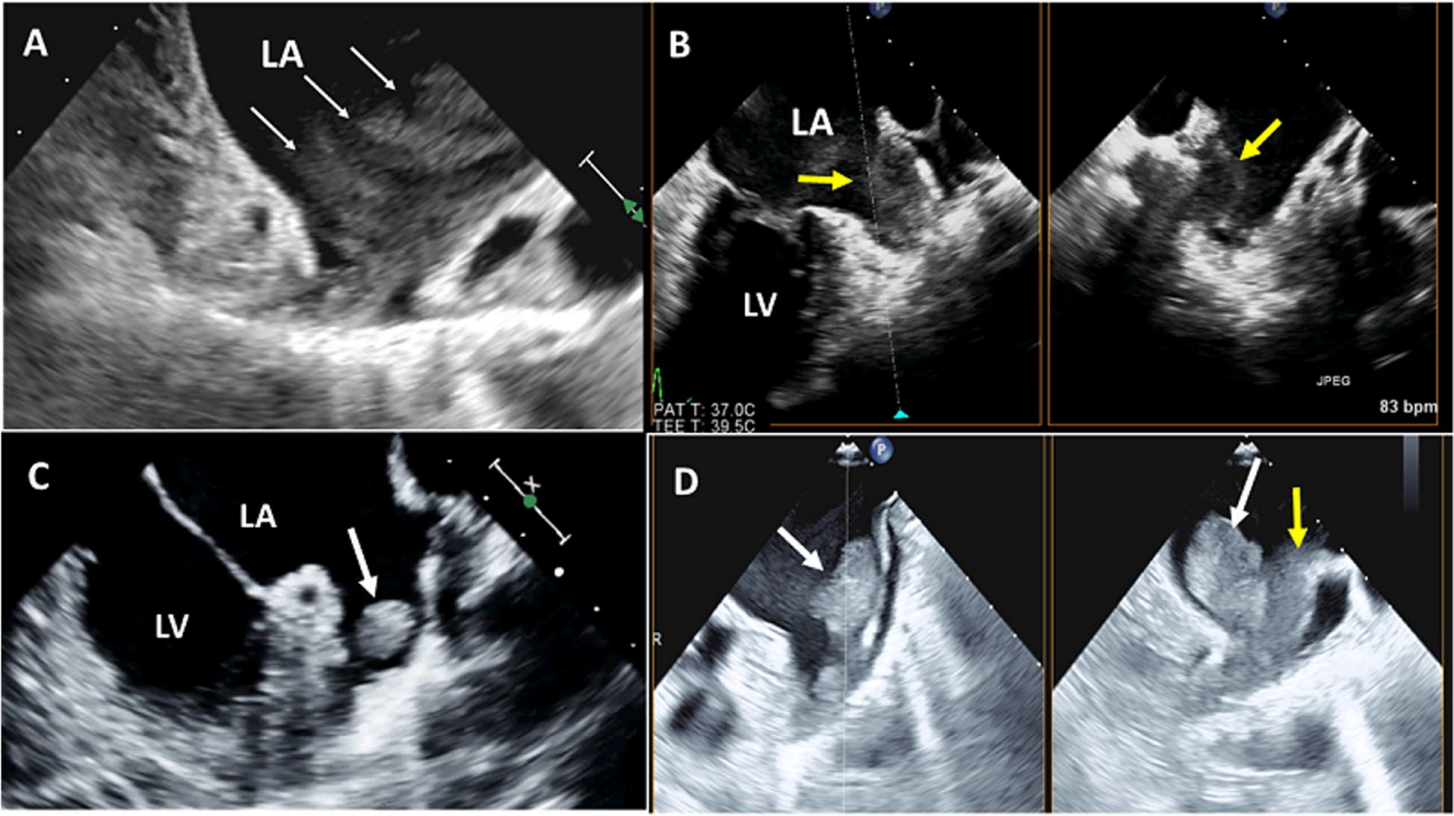
Left atrial appendage (LAA) visualization by two-dimensional transesophageal echocardiography shows the sequel of blood stasis. **A** Severe spontaneous echo contrast (small white arrows). **B** X-plane view of LAA shows dense spontaneous echo contrast associated with sludge formation (yellow arrow). **C** Small rounded LAA thrombus (white arrow). **D** Large LAA thrombus protruding into the LA (white arrows) associated with sludge (yellow arrow)

This symptom is present in up to 60% of all patients with AF and in more than 80% of those with LAA thrombi or a recent thromboembolic event. Patients with dense SEC have a stroke rate of 18.2% per year if not treated with warfarin, compared to 4.5% per year when on warfarin [20]. On TEE imaging, SEC appears as a smoke-like swirling pattern. However, gain adjustment is needed to visualize it and distinguish it from white noise artifacts. The SEC severity was graded semiquantitatively from 0 to 4, as follows [21]:

- 0: No echogenicity.
- 1 + (mild): Minimal echogenicity that is imperceptible at normal gain settings but may be detectable transiently with optimal gain during the cardiac cycle and is located in the LAA or sparsely distributed in the LA cavity.
- 2 + (mild to moderate): A denser swirling pattern that is detectable with normal gain settings and has a distribution similar to that of 1 + .
- 3 + (moderate): A dense swirling pattern in the LAA that generally is associated with a relatively lower intensity throughout the main LA cavity and may fluctuate in intensity but is detectable constantly throughout the cardiac cycle.
- 4 + (severe): Intense echogenicity and very slow swirling patterns in the LAA, usually with a similar density in the main LA cavity (Fig. 4A).

### LAA sludge

This is a stage between SEC and thrombus formation (denser than SEC, less dense than a thrombus). It has a dynamic, viscous, gelatinous morphology that is not well formed, without a discrete mass visualized throughout the cardiac cycle (Fig. 4B) [22].

### LAA *thrombus*

Using TEE, a thrombus is visualized as a mobile echo density within the LAA that is distinct from the pectineal muscles and moves independently of the LA walls. Due to the complex structural features of the LAA, overdiagnosis (artifacts, trabeculae, or prominent pectinate muscles) and underdiagnosis (hidden thrombi in a multilobed LAA) are not uncommon (Fig. 4C, D).

### Indices of a dysfunctional LAA

There is no standard normal reference value for LAA size and function. However, the indicators of LAA dysfunction from the published data include the following [23–25]:

- LAA emptying velocity < 20 cm/sec is associated with SEC and thrombi formation.
- Enlarged LAA: LAA volume index > 5.6 mL/m^2^.
- The cutoff values of LAA end-diastolic and end-systolic volumes in thrombosis of the LAA were 18.45 and 9.69 mL, respectively.
- LAA-EF using < 37.5% best predicted SEC or thrombus in patients with valve disease.

## Echocardiographic modalities and practical tips

Integration of both conventional and novel echocardiographic modalities is essential to obtain a full morphological and functional assessment of the LAA.

## 2D-TTE

**Fig. 5:**
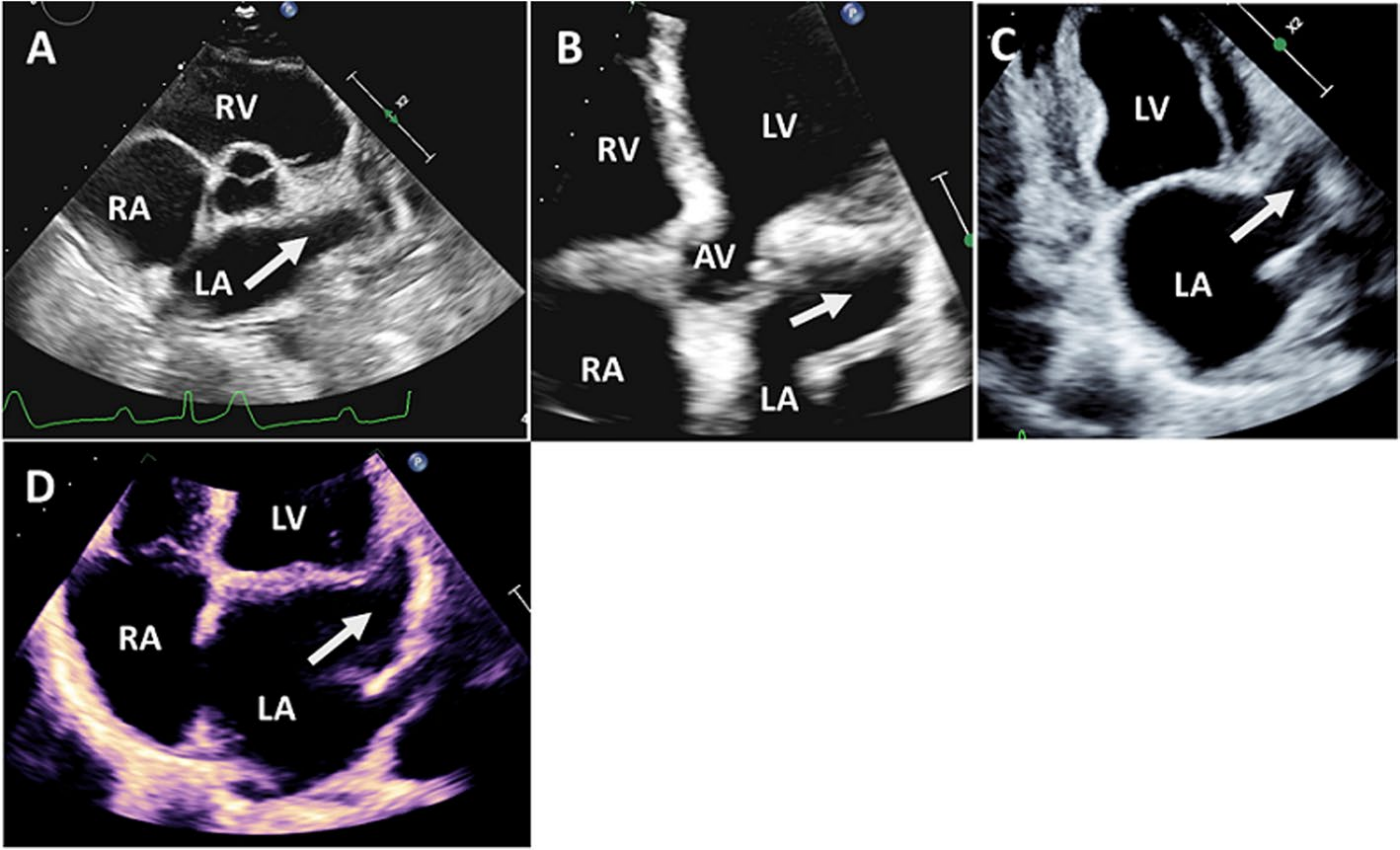
Visualization of the left atrial appendage (white arrow) using two-dimensional transthoracic echocardiography. **A** From the parasternal short-axis view at the aortic valve level. (**B**) Apical five-chamber view. **C** Apical two-chamber view. **D** Apical four-chamber view

In most cases, it is too difficult to obtain ideal quality images of the LAA using 2D-TTE alone due to its small size, distant position from the transducer, and limited echo windows. Thus, 2D-TTE is limited in its ability to evaluate the morphology and function of the LAA and to determine the presence or absence of thrombi. The standard views of the LAA include the parasternal short-axis view at the level of the aortic and pulmonic valves with a slight clockwise rotation or downward tilt of the transducer, an apical five-chamber view with upward tilting of the transducer, and an apical two-chamber view with a slight lateral tilt or clockwise rotation of the transducer (Fig. 5). Harmonic imaging can improve LAA visualization, especially in cases of an enlarged LA. Administration of contrast agents has enhanced the capability of 2D-TTE to detect LAA thrombi [26].

## 2D-TEE

**Fig. 6:**
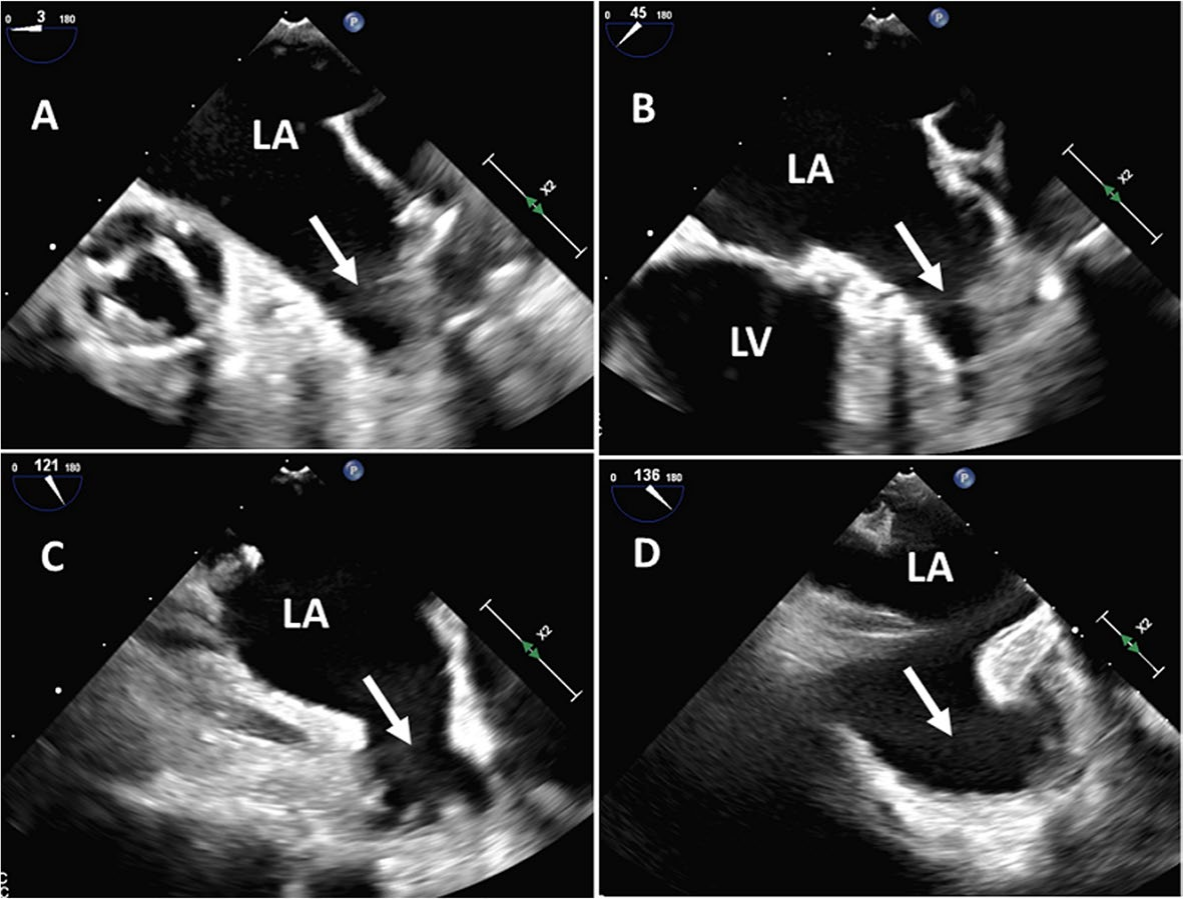
Visualization of the left atrial appendage (white arrow) using two-dimensional transesophageal echocardiography from the mid-esophageal level at different angles. **A** Between 0°–20°. **B** Between 45°–60°. **C** Between 80°–100°. **D** Between 120°–135°

A 2D-TEE is considered the gold standard for assessing and studying LAA morphology and anatomy because of the close proximity of the transducer to the LAA, which allows excellent imaging. Compared with intraoperative observations, 2D-TEE has high sensitivity (92%) and specificity (98%) with negative predictive value of 100% and positive predictive value of 86% [27]. Visualization of the LAA can be accomplished easily first in the mid-esophageal aortic valve short-axis view (30°–60°) and then by anteflexing the transducer with an angle rotation from 0° to 180°. The standard views usually acquired from the mid-esophageal level to visualize the LAA are as follows (Fig. 6): 0° to 20° in the four-chamber view modified by slight flexion or withdrawal to open the LAA; 45° to 60° at the level of the aortic valve; 80° to 100° in the apical two-chamber view; 120° to 135° in the long-axis view, turned anticlockwise to open subsidiary lobes within the LAA. In addition, 130° to 180° is a reverse boot view to enhance LAA trabeculations.

Using 2D-TEE, the LAA is visualized only in one plane at any given time, and it is not possible to obtain simultaneous multiple views and planes. This limitation interferes with accurate measuring of the LAA opening, depth, and area and leads to a biased estimation of LAA size. In addition, acoustic shadowing of the coumadin ridge and slight transverse sinus pericardial fluid accumulation may be confused with LAA thrombi. In real practice, the diagnostic accuracy of 2D-TEE may not be as high as predicted because of both false-positives (pectinate muscles labeled as thrombi) and false-negatives (small thrombi < 2 mm hidden in one of the lobes) [28].

## 3D-TEE

A 3D-TEE imaging can preserve spatial and temporal resolution and overcome some of the limitations of 2D-TEE (such as inadequate imaging planes). Through the number of sagittal and coronal planes and multiple functions (live 3D, full volume, zoom, TrueVue, and TrueVue Glass), 3D-TEE allows a comprehensive assessment of the complex morphology of the LAA and the surrounding structures to improve stroke prediction in AF [29]. Compared with computed tomography, 3D-TEE demonstrated higher accuracy and reliability than 2D-TEE in the assessment of thrombus shape, size, mobility, and structure, with a significantly lower false positive rate of diagnosis [30].

However, data remain limited regarding the sensitivity and specificity of 3D-TEE. Acquisition of a full volume 3D dataset enables measurements of the LAA orifice area, depth, and volume, all of which correlate well with those obtained by computed tomography. During procedures, 3D-TEE has become important to guide LAA closure due to lesser interobserver and intraobserver variability and better agreement with the implanted device compared to those of 2D-TEE [31].

### Intracardiac echocardiography

Intracardiac echocardiography **(**ICE) is a feasible and safe alternative to avoid general anesthesia and the associated potential risks of TEE. ICE and TEE appear to have equivalent diagnostic efficacy to determine LAA dimensions, rule out a LAA thrombus, and verify the location and stability of occluder devices [32]. For LAA occlusion, the ICE-guided procedure was not inferior to the TEE-guided procedure regarding feasibility, safety, and efficacy [33]. Compared to 2D-TEE, the optimal ICE imaging strategy and views for LAA imaging have not been defined, and the clinical experience among echocardiographers is limited. Visualization of the LAA within the LA requires a trans-septal puncture. The ICE catheter tip can be placed in the following structures [34]:

- LA body: The ICE catheter tip can be positioned centrally in the LA, aiming toward the LAA so that the edge of the LA ridge can be visualized, and separating the LAA from the left upper pulmonary vein.
- Left upper pulmonary vein: Advancing the ICE catheter tip deeper into the left upper pulmonary vein allows a long-axis view of the LAA.
- Left lower pulmonary vein: Placing the ICE tip in the left lower pulmonary vein with slight angulation could display the LAA lobe structure.
- Transmitral: In a transmitral placement, the ICE catheter is deflected and advanced across the mitral valve with the ultrasound beam pointing superiorly, visualizing the LAA.

Images can be obtained from the right atrial cavity without septal puncture. However, images of the LAA are susceptible to attenuation by many structures such as a thick interatrial septum, LA wall, prosthetic valve, calcified aortic valve, calcified MV, or calcified annulus. The tip of the ICE probe can be placed in the following structures:

- Coronary sinus: Advancing the probe inside the coronary sinus with gentle rotation to center the LAA image usually provides excellent LAA images.
- Para coronary sinus view: The para coronary sinus view is obtained by rotating the catheter counterclockwise and steering it posteriorly to place the tip just below the coronary sinus ostium.
- Pulmonary artery or right ventricular outflow tract: Advancing the ICE probe into the pulmonary artery allows good visualization of the LAA due to their proximity.

### 3D/4D-ICE

The advancement of matrix technology enabled the advent of a new generation of ICE transducers with a 90° × 90° 3D field of view. The novel 3D-ICE catheter is capable of real-time volumetric imaging with both 2D and 3D color Doppler flow. The catheter allows multiplanar visualization of target cardiac structures with minimal catheter manipulation [35]. Currently available 3D-ICE catheters are AcuNav Volume by Siemens, VeriSight Pro by Philips, and Biosense Webster NuVision using GE Healthcare Vivid Ultra Edition ultrasound systems. The available clinical studies showed good diagnostic and procedural guidance of 3D-ICE in radiofrequency, cryoablation, and LAA occlusion procedures [35, 36]. Currently, the real-world experience of 3D/4D-ICE use is limited due to its cost. In addition, ICE imaging is inferior to the sector size of 3D-TEE. Development of more advanced catheter technology will enhance image acquisition and increase use of ICE imaging to many interventional procedures.

### Contrast echocardiography

In some cases, it may not be possible to differentiate a thrombus from the pectinate muscles or artifacts especially with sluggish flow, presence of SEC, or poor visualization. The use of ultrasound contrast with power Doppler imaging, especially during TEE, achieves complete opacification of the LAA and confirms or excludes the presence of a LAA thrombus [37].

### Pulsed wave Doppler

Pulsed wave Doppler is used to record the LAA waves through optimal alignment of the Doppler signal with the LAA flow using color flow imaging. The position of the sample volume at the site of maximal flow velocities usually is in the proximal third or < 1 cm from the LAA orifice. The gain should be low, and care must be taken to avoid artifacts produced by inadvertent sampling of the LAA wall. An average of five cardiac cycles has to be considered in AF [14, 15].

### Color Doppler imaging

Color Doppler imaging can be used in the identification of LAA thrombi as areas with decreased or absent color flow within the LAA.

### Tissue Doppler imaging

The LAA tissue velocity can be obtained as a direct measurement of LAA contractile function [16]. The LAA tissue velocity is acquired from the LAA long-axis view with a sample size of 2.5 mm placed at the lateral and septal wall. Some studies showed that LAA tissue velocity has good feasibility and correlates with LAA dysfunction in patients with mitral stenosis, hypertension, and hypertrophic cardiomyopathy [38]. However, tissue Doppler is affected by the tethering and translation effects of the heart and is not a true measure of myocardial contractility.

### Speckle-tracking echocardiography

STE overcomes the limitations of tissue Doppler. Using STE, LAA strain measurement is feasible. Reproducible parameters are employed to quantify LAA contractile function in sinus rhythm and in AF. Reduced global longitudinal (reservoir, conduit, and atrial contractile) and regional strain rates correlate positively with LAA filling and emptying velocities in AF patients and in patients after cardioversion [39]. The global LAA strain was low in paroxysmal nonvalvular AF and even lower in persistent AF. Regional and global LAA strain is depressed significantly in AF with SEC/thrombus [40]. To measure the LAA longitudinal strain and strain rate, at least three cardiac cycles must be assessed from the mid-esophageal two-chamber view for offline analysis. Tracing of the medial wall starts from the beginning of the medial part of the LAA opening to the LAA apex, while that of the lateral wall starts from the beginning of the lateral part of the LAA opening to the LAA apex (Fig. 3E) [41]. LAA mechanical dispersion (the standard deviation of the time to peak positive strain corrected by the R-R interval) was addressed as an independent determinant of LAA SEC/thrombus [42].

### Step approach protocols for LAA assessment

Echocardiographic assessment of the LAA is indicated in many clinical situations. For each indication, there is specific information and certain parameters to guide management.

### Stroke workup

Cardioembolic strokes account for ≥ 15% of all ischemic strokes. In approximately 75% of patients, the emboli arise from the LAA (90% in nonrheumatic AF and 60% in rheumatic MV disease). An LAA thrombus increases the risk for transient ischemic attacks, cerebrovascular accidents, and strokes. In such patients, the presence of SEC alone without thrombus may be enough to diagnose a cardioembolic stroke. Assessment of the LAA must include analysis of both morphologic parameters and hemodynamic indices for risk stratification [43]. The following specific predictors of stroke have been suggested by multiple studies: low-peak LAA velocity (< 20 cm/sec), LAA orifice area, and LAA morphology (4% in chicken wing, 10% in windsock, 12% in cactus, and 18% in cauliflower) [44, 45]. All these predictors have to be considered during a stroke workup regardless of the presence or absence of an LAA thrombus. In patients with an embolic stroke of undetermined source without known AF, high-risk LAA morphology was described in many studies as non-chicken-wing morphology, shallow depth, and narrow orifice diameter [46, 47]. In addition to LAA morphology, LAA reservoir strain was a significant predictor of subclinical AF (80% sensitivity and 73% specificity) [48]. Malignant LAA is a term used for AF patients who experienced stroke and/or who are diagnosed with LAA thrombus despite full anticoagulation. The optimal treatment strategy to reduce the embolic risk in these patients remains controversial [49].

### Before AF cardioversion

Once AF occurs, LA and LAA lose their active contraction, which leads to failure of effective ejection and eventual formation of SEC/thrombus. Before AF cardioversion, 2D-TTE should be performed to define the type of AF (valvular and nonvalvular). Identifying the type of AF helps to identify the risk markers for LAA thrombosis and to predict the success of rhythm control [50]. In patients with AF > 48 h onset and/or when the exact duration and onset of AF cannot be determined, the current guidelines recommend anticoagulant therapy for at least 3 weeks before and 4 weeks after cardioversion. The guidelines still recommend TEE before an elective cardioversion as an alternative in patients who do not meet the requirement of uninterrupted effective oral anticoagulation for ≥ 3 weeks. If the thrombus is identified, TEE should be repeated after 3 to 4 weeks of anticoagulation [51].

### After cardioversion

The phenomenon of LAA “stunning” was demonstrated in a series of patients who suffered post-cardioversion strokes despite the absence of an LA or LAA thrombus on pre-cardioversion TEE. LAA “stunning” has been explained as a temporary paradoxical reduction in LAA flow velocities and worsening mechanical function of both the LA and the LAA [52]. This phenomenon is evidenced by increase in the SEC intensity and decrease in LAA Doppler flow velocities immediately after cardioversion to sinus rhythm. Atrial stunning gradually improves over a course of days to weeks depending on the duration of AF [53]. The predictors of successful cardioversion and maintenance of sinus rhythm have been suggested as follows.

- LAA flow velocity (late diastolic emptying velocity) < 33.9 cm/sec was associated independently with AF recurrence and stroke after successful electric cardioversion. A velocity < 20.2 cm/sec was associated with mortality [54].
- LAA emptying velocity > 40 cm/sec was associated significantly with maintenance of sinus rhythm following cardioversion at 1 year, which was independent of the SEC, LA dimension, LV ejection fraction, and duration of AF < 1 week [55].
- LAA tissue velocity > 8 cm/sec was correlated with successful cardioversion (sensitivity 70% and specificity 63%) [56].

### Pulmonary vein isolation

In AF, the LAA neck becomes rounder due to stretching with loss of LAA compliance and contractility. Successful radiofrequency ablation to AF will significantly decrease the orifice, neck, and length of the LAA and reverse the LAA neck shape. Many studies have suggested that ablation of the LAA anterior wall near its neck and electrical isolation can effectively prevent AF recurrence. The combined model of LAA morphology, orifice area, volume index, LAA-EF, and LAA emptying velocity was an effective predictor of recurrence following radiofrequency ablation [57, 58].

### LAA occlusion

**Fig. 7:**
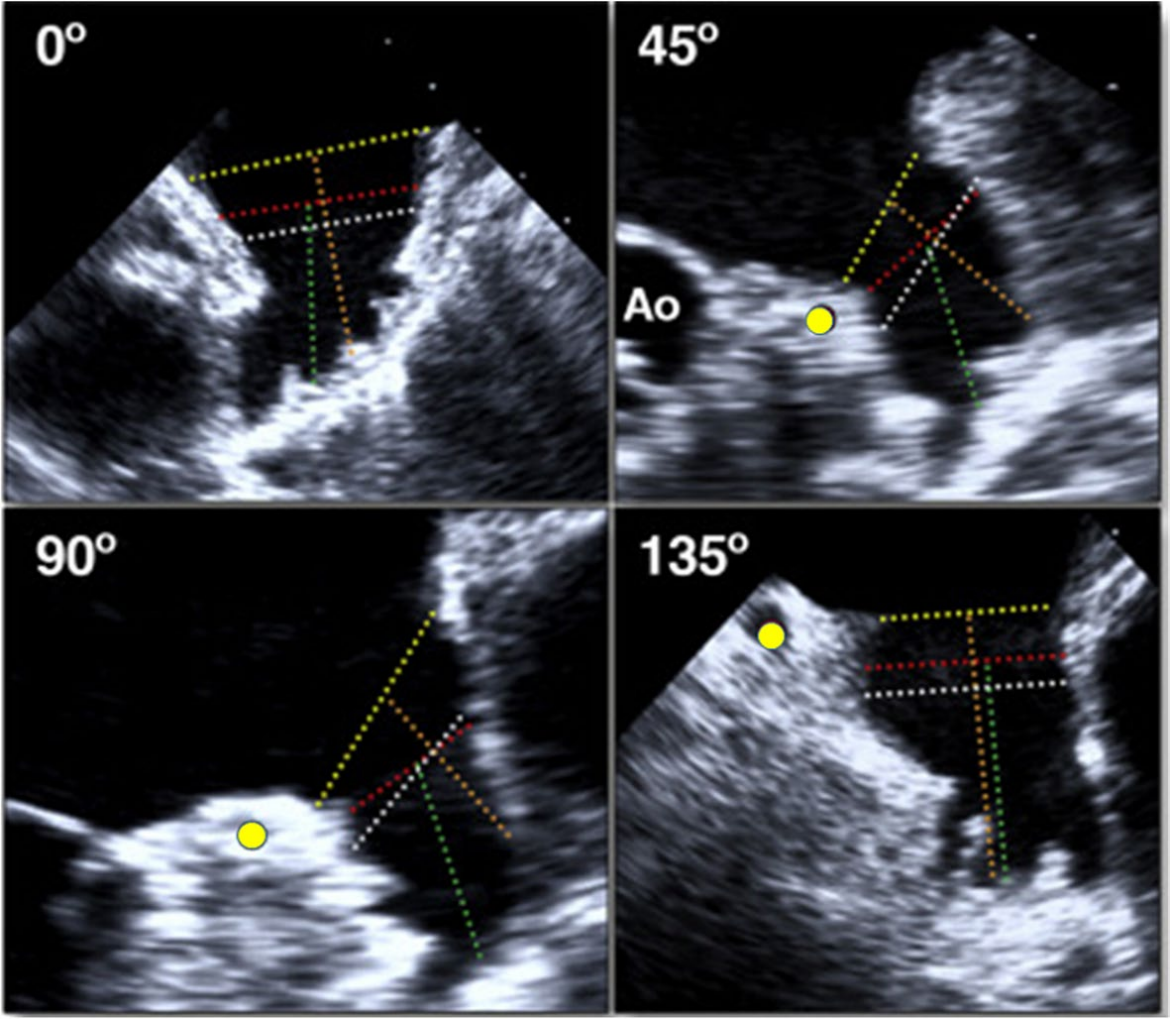
Scan of the left atrial appendage from 0°, 45°, 90°, and 135° views using two-dimensional transthoracic echocardiography to measure the maximum orifice diameter, landing zone, and maximum depth before device closure. Yellow circle indicates the circumflex artery

In patients with nonvalvular AF and contraindications to oral anticoagulation, percutaneous LAA occlusion by placement of an intravascular device into the LAA may reduce the stroke risk (Fig. 7A). A large European registry reported a high implantation success rate (98%) with an acceptable procedure-related complication rate of 4% at 30 days [59]. The long-term outcomes included a reduced annual risk of stroke by 84% and reduced annual risk of bleeding by 27% [60]. Over the past 15 years, multiple percutaneous LAA occlusion devices have been developed, with significant improvement in peri-procedural results over time. In all stages of the procedure (pre-, peri-, and post-procedure), TEE is the gold standard modality [61].

#### Pre-procedure

Once a thrombus has been excluded, volume loading should start to increase the LAA size, which improves device selection and outcome. Scanning the LAA from 0°, 45°, 90°, and 135° views using both 2D- and 3D-TEE is essential for proper device selection (Fig. 7) [62, 63] and should involve the following.

- LAA morphology including the number of lobes and the lobe positions relative to the ostium.
- The maximum LAA orifice diameter from the level of the circumflex coronary artery up to a point 1 to 2 cm from the tip of the left superior pulmonary vein limbus. This measurement can be obtained in the LAA long-axis view at the MV level or at the aortic valve level. The orifice perimeter and area can be measured using 3D-TEE software analysis.
- The LAA landing zone using 3D-TEE multiplanar reconstruction with alignment of the orthogonal planes at the proposed landing zone differs from one device to another. For example, the landing zone for the Amplatzer is approximately 10 mm distal from the ostial plane into the lobe, while that for the Watchman is at the level of the circumflex coronary artery to a point 1 to 2 cm distal to the tip of the ligament of Marshall.
- The maximum depth of the dominant LAA lobe.
- Left upper pulmonary vein diameter and peak systolic and diastolic flow velocities.
- Selection of the proper device depends on the pre-procedural 2D/3D-TEE assessment of the LAA. The device size is chosen based on the manufacturer’s sizing guide in the instructions for use. Correct sizing of the devices is important to avoid leakage around the edges of the device (with undersizing) and compression of the circumflex artery and of LAA perforation (with oversizing).

#### Peri-procedure

**Fig. 8:**
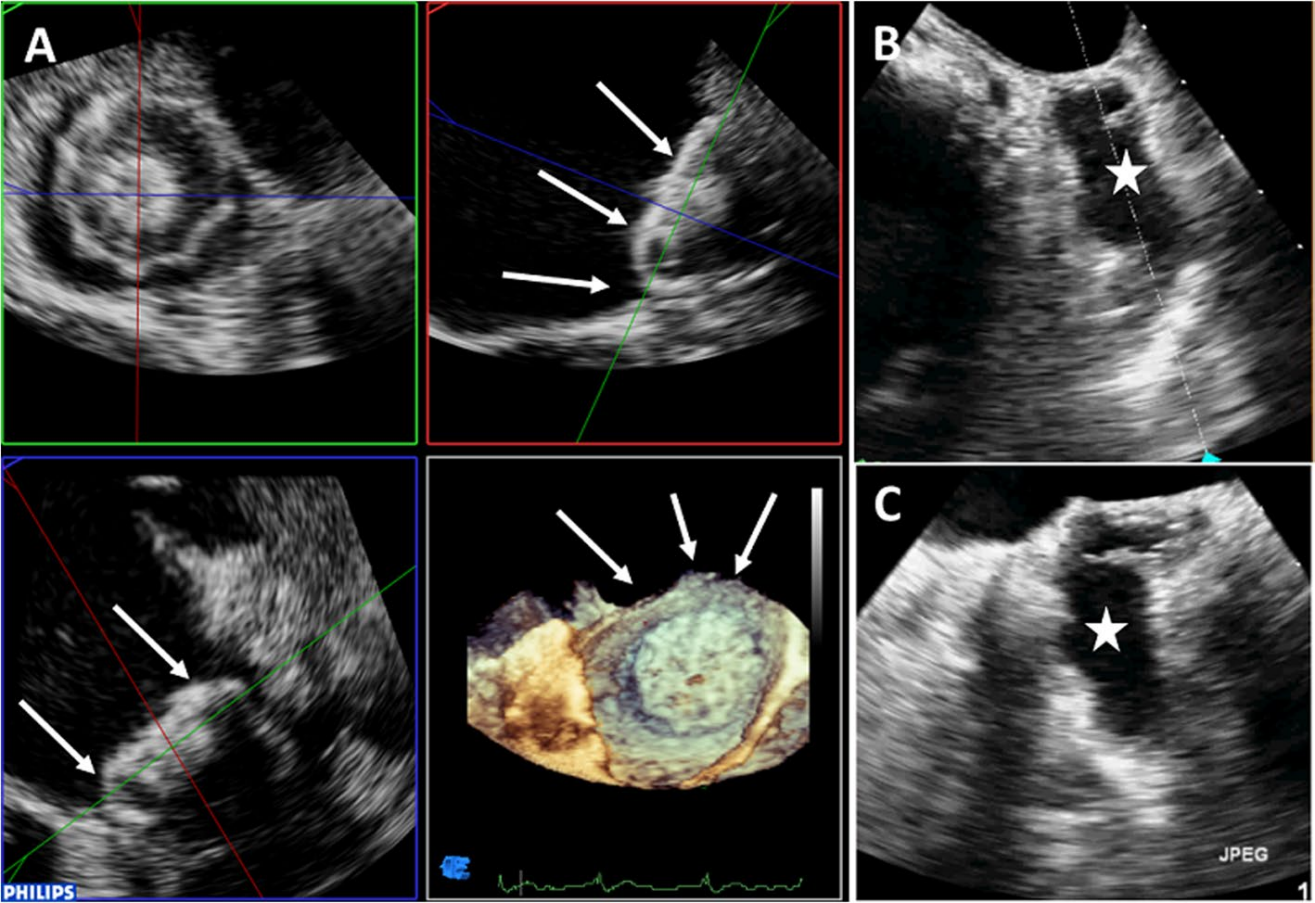
F (**A**) Quad-screen acquisition using three-dimensional transthoracic echocardiography shows a Watchman (Boston Scientific) closure device of the left atrial appendage (white arrows). **B**, **C** Left atrial appendage closed surgically (star)

Due to the 3D-TEE ability to visualize the moving wires, catheter, and devices inside the LA and LAA, its role in the procedural guidance is to do the following (Fig. 8) [64]:

- Guide the septal puncture at the proper site (posteriorly and inferiorly).
- Confirm the coaxial guide trajectory prior to device delivery.
- Determine appropriate implantation depth and device stability using the tug test.
- Check device compression prior to its release; 8% to 20% compression is recommended. Low compression is associated with embolization risk and peri-device leaks, while overcompression can lead to erosion or rupture of the LAA.
- Ensure that the device does not protrude > 4 to 7 mm beyond the LAA ostium.
- Exclude significant peri-device leaks (defined as jet width of 1 to 5 mm) using color Doppler with a lowered Nyquist limit.
- Exclude encroachment on surrounding structures, particularly the left superior pulmonary vein (diameter and velocity) and MV (disruption of transmitral flow).

#### Post-procedure

2D-TEE is required to exclude complications such as pericardial effusion and new LV wall motion abnormalities in the territory of the circumflex artery due to its proximity to the LAA ostium. Follow-up (at 1 and 6 months and annually post-procedure) the following: device position, migration, displacement, erosion; device-related thrombi or fibrosis inside the LAA; and persistence of significant peri-device leak (> 5 mm).

### LAA exclusion

Many surgical techniques are used for LAA exclusion including amputation and closure, stapler closure, and double-layer linear closure from within the atrium (Fig. 7B, C). The current guidelines provide class II recommendations for LAA exclusion during cardiac surgery because much of the data on feasibility and safety is observational and nonrandomized [65]. Recently, the LAAOS III (Left Atrial Appendage Occlusion Study) multicenter randomized trial included 4,811 patients who underwent cardiac surgery and had AF and CHA_2_DS_2_-VASc (congestive heart failure, hypertension, age ≥ 75 years [doubled], diabetes, stroke [doubled], vascular disease, age 65–74 years, and sex category [female]) scores ≥ 2. The patients were randomized to receive the standard procedure or to undergo concomitant LAA occlusion. At 3-years of follow-up, the LAA occlusion group had a 33% lower risk for stroke or systemic embolism than did those who received standard care [66]. LAA exclusion can be performed as a minimally invasive epicardial catheter approach or thoracoscopic clipping. Currently, three devices have US Food and Drug Administration approval for transpericardial LAA closure: Lariat (SentreHEART), AtriClip (AtriCure), and Tiger Paw II (Maquet Cardiovascular) [67]. The field continues to evolve, and newer techniques may offer higher rates of complete LAA occlusion. Before delivery of an epicardial LAA suture exclusion device, 3D-TEE is useful to guide a complete snare around the body of the LAA. Evidence of complete occlusion by TEE should be obtained before cessation of oral anticoagulant therapy because incomplete surgical ligation is associated with a significant increase in thromboembolism [68].

## Conclusions

Echocardiography is an essential tool in the assessment of LAA in many indications. It provides important diagnostic and prognostic information that helps with proper management. Integration of both conventional and modern echocardiographic techniques is valuable during interventions for better selection, guidance, and outcome assessment.

## Data Availability

Not applicable.

## Abbreviations

AF: Atrial fibrillation
CHA2DS2-VASc: Congestive heart failure, hypertension, age ≥ 75 years (doubled), diabetes, stroke (doubled), vascular disease, age 65–74 years, and sex category (female)
D: Dimensional
ICE: Intracardiac echocardiography
LA: Left atrial
LAA: Left atrial appendage
LAA-EF: LAA volume and emptying fraction
LAAOS III: Left Atrial Appendage Occlusion Study
LV: Left ventricular
MV: Mitral valve
SEC: Spontaneous echo contrast
STE: Speckle-tracking echocardiography
TEE: Transesophageal echocardiography
TTE: Transthoracic echocardiography
